# Fatty Acid Profile and Genetic Variants of Proteins Involved in Fatty Acid Metabolism Could Be Considered as Disease Predictor

**DOI:** 10.3390/diagnostics13050979

**Published:** 2023-03-04

**Authors:** Raja Chaaba, Aicha Bouaziz, Asma Ben Amor, Wissem Mnif, Mohamed Hammami, Sounira Mehri

**Affiliations:** 1 Lab-NAFS “Nutrition-Functional Food & Health”, Faculty of Medicine, University of Monastir, Avicene Street, Monastir 5000, Tunisia; 2 Higher School of Health Sciences and Techniques, Sousse, University of Sousse, Sousse 4054, Tunisia; 3 Bio-Resources, Integrative Biology & Valorization (BIOLIVAL, LR14ES06), Higher Institute of Biotechnology of Monastir, University of Monastir, Monastir 5000, Tunisia; 4 Faculty of Medicine, “Ibn El Jazzar” University of Sousse, Sousse 4054, Tunisia; 5 Department of Chemistry, Faculty of Sciences, University of Bisha, P.O. Box 199, Bisha 61922, Saudi Arabia

**Keywords:** fatty acids, diseases, gene polymorphisms, plasma, red blood cells

## Abstract

Circulating fatty acids (FA) have an endogenous or exogenous origin and are metabolized under the effect of many enzymes. They play crucial roles in many mechanisms: cell signaling, modulation of gene expression, etc., which leads to the hypothesis that their perturbation could be the cause of disease development. FA in erythrocytes and plasma rather than dietary FA could be used as a biomarker for many diseases. Cardiovascular disease was associated with elevated trans FA and decreased DHA and EPA. Increased arachidonic acid and decreased Docosahexaenoic Acids (DHA) were associated with Alzheimer’s disease. Low Arachidonic acid and DHA are associated with neonatal morbidities and mortality. Decreased saturated fatty acids (SFA), increased monounsaturated FA (MUFA) and polyunsaturated FA (PUFA) (C18:2 n-6 and C20:3 n-6) are associated with cancer. Additionally, genetic polymorphisms in genes coding for enzymes implicated in FA metabolism are associated with disease development. FA desaturase (FADS1 and FADS2) polymorphisms are associated with Alzheimer’s disease, Acute Coronary Syndrome, Autism spectrum disorder and obesity. Polymorphisms in FA elongase (ELOVL2) are associated with Alzheimer’s disease, Autism spectrum disorder and obesity. FA-binding protein polymorphism is associated with dyslipidemia, type 2 diabetes, metabolic syndrome, obesity, hypertension, non-alcoholic fatty liver disease, peripheral atherosclerosis combined with type 2 diabetes and polycystic ovary syndrome. Acetyl-coenzyme A carboxylase polymorphisms are associated with diabetes, obesity and diabetic nephropathy. FA profile and genetic variants of proteins implicated in FA metabolism could be considered as disease biomarkers and may help with the prevention and management of diseases.

## 1. Introduction

Fatty acids (FA) belong to the lipid class. They can be free or associated with alcohols to provide triglycerides, phospholipids, cerids or sterides. They can be saturated (SFA) or unsaturated (UFA) depending on the presence or absence of a double bound in their structure. Based on the number of double bounds, we can distinguish between monounsaturated FA (MUFA with only one double bound) or polyunsaturated FA (PUFA with two or more double bounds). According to the carbon chain length, four groups of FA are identified: short-chain FA with 4 to 6 carbon atoms, medium-chain FA with 8 to 12 carbon atoms, long-chain FA with 14 to 20 carbon atoms, and very long-chain FA with 22 or more carbon atoms. Depending on the configuration, a distinction is made between trans FA and cis FA. FA differ from each other in the number of carbon atoms, unsaturations and amount of configuration. They are present in all cells and tissues of the body. SFA and MUFA are synthesized by all organisms. However, not all PUFA are synthesized by mammals, including humans, because they do not have the enzymes (Δ12- and Δ15 desaturases) necessary for their synthesis. These are therefore called essential FA. These FA are represented by alpha-linolenic acid (ALA, C18: 3 n-3) [1] and linoleic acid (LA, C18: 2 n-6) [2]. They will play the role of precursors of other FA with longer chains and of bioactive mediators in the form of oxygenated molecules (eicosanoids, docosanoids, etc.) [3].

### 1.1. Different Fatty Acids in Plasma and Erythrocytes

Lipids are characterized by a high proportion of phospholipids, while reserve lipids mainly take the form of triglycerides. Phospholipids and triglycerides are both made up of FA. 

Fatty acids are multiple and variable. Their profile is not constant in all species. UFAs are more common in plants, while SFAs are more common in animals. Additionally, FA differ between individuals in the same species. These differences can be explained by variation in dietary habits or by genetic differences that may affect the proteins involved in FA metabolism [4,5]. However, according to different studies, in all people, palmitic acid is the most frequent SFA, oleic acid is the most frequent MUFA and LA is the most frequent PUFA (Table 1) [6,7,8,9]. 

Fatty acid analyses are often conducted on samples of whole blood or plasma or erythrocytes (RBC). In fact, plasma analyses reflect recent FA intake obtained through the diet, while analyses of RBC and adipose tissue reflect dietary fatty acids intake over the long term [10]. The results of selected three studies in three different populations (Tunisia, Chile and the USA) exploring both profiles of plasma and erythrocyte clearly show how these profiles differ, comparing the percentage abundances of individual FA. Total saturated FA mainly stearic acid are more frequent in RBC, whereas linoleic acid is more frequent in plasma.

Indeed, the FA profile is not the result of dietary intake alone; several other factors can intervene, such as genetic factors [5]. 

### 1.2. Functions of Fatty Acids

FA are highly energetic substrates. In the mitochondria, they provide a large amount of energy through beta oxidation. This quantity depends on the number of carbons that make up the FA, but it is always greater than that provided by a molecule of glucose (for FA with more than four carbons).

FA are the main constituent of cell membranes. They influence membrane fluidity [11]. Incorporated into membrane phospholipids, PUFA increase membrane fluidity and can affect cell function. Moreover, the FA composition of the membrane influences cell signaling. Increasing the concentration of docosahexaenoic acid (DHA, C22:6 n-3) in the excitable membranes of the brain and retina acts on cell signaling by altering lipid rafts at this level [12]. Impaired insulin signaling by free FA leads to endothelial dysfunction [13]. MUFA affect the Intracellular Signaling in Cancer [14].

FA can be precursors of oxygenated molecules. Dihomo-gamma-linolenic (C20:3 n-6), arachidonic (AA, C20:4 n-6), and eicosapentaenoic (EPA, C20:5 n-3) acids enter the enzymatic pathways of oxygenation, hydroxylation and of peroxidation to form eicosanoids (powerful mediators). Membrane FA released under the effect of phospholipase A2 can enter two different metabolic pathways: (i) the cyclo-oxygenase (COX) pathway which will give rise to prostaglandins, prostacyclins and thromboxanes and (ii) the lipoxygenase (LOX) pathway, which produces leukotrienes and hydroperoxidized FA [3]. In addition, peroxidation of 20-carbon PUFAs through the cytochrome P450 pathway can yield epoxyeicotrienoic acids. AA and DHA can yield isoprostanes and neuroprostanes through non-enzymatic peroxidation [3].

PUFAs have the ability to bind directly or through their derivatives (eicosanoids) to transcription factors, which modulate the expression of certain genes involved in various metabolic pathways [15]. The most important transcription factors are peroxisome proliferator-activated receptors (PPARs), sterol regulatory element binding protein (SREBP-1c for sterol regulatory element binding protein-1c), hepatic nuclear factors (HNF4 for hepatic nuclear factor 4), retinoid receptor (RXRa for retinoid X receptor) and the hepatic X receptor (LXRa for liver X receptor).

Given the important roles played by FA, it can be assumed that an abnormal lipid profile will be associated with disease development or prevention in different ways. In fact, FA can influence inflammation, oxidation and immunity [16]. For example, LA is a precursor of AA, which is a precursor of certain factors involved in inflammation, such as prostaglandins and leukotrienes. This leads us to assume that a high level of these FA will be accompanied by significant inflammation. However, this hypothesis should be rejected [17]. However, these FA can intervene in different ways in inflammation: (i) acting (inhibiting or activating) on the Toll-like receptor4 signaling pathways [18]; (ii) acting on the expression of microRNAs [19]. Moreover, FA are implicated in oxidation in different manners (i) they are involved in membrane lipid peroxidation, which induces Ferroptosis [20]; (ii) they are associated with LDL oxidation [21]; (iii) they have an impact on redox status [22].

## 2. Materials and Methods

PubMed database searches were performed to search for articles related to this subject. For the paragraphs about fatty acid characteristics in many diseases, we introduced fatty acids and disease prediction as key words and 10 years as the filter. For the association between gene polymorphisms and disease, the keywords used to query the database were desaturase polymorphism, elongase polymorphism, Fatty Acid Binding Protein polymorphism, and Acetyl-coenzyme A carboxylase polymorphism. The number of items found was very large. The preliminary selection of articles used in this review was based on the content of the abstracts. The abstracts needed to be consistent with the objective of the present work: the fatty acids had to be analyzed in blood and not in other tissues, nor in food or food supplements. After this step, reading the articles allowed us to select the articles that took the greatest number of fatty acids into consideration. Articles based on the study of a single fatty acid were excluded. In all articles, FA were measured in total lipids and not in lipid subfraction (phospholipids, ceramide, cholesterol ester, etc.).

## 3. Results and Discussion

### 3.1. Fatty Acids and Diseases

Dietary fat intake is associated with the development of many diseases. Since 1970, reduced saturated fat consumption has been recommended in the US [23]. A diet with high PUFA and MUFA and with low SFA and trans FA prevents cardiovascular disease [24,25]. In fact, FA in plasma, RBC and adipocytes reflect dietary FA. However, this review will not focus on the relationship between dietary FA and diseases, but will focus on fatty acid assays in humans (in plasma, RBC) in order to answer the following question: can we consider individual FA as biomarkers of many diseases?

#### 3.1.1. Fatty Acids and Cardiovascular Disease (CVD)

FA play a crucial role in CVD. They exert their effect by acting on lipoprotein metabolism [26] and endothelial function [27]. FA-mediated dysregulation of nitric acid and cytokine production, inflammation, oxidative stress, apoptosis, and activation of the renin–angiotensin system, which causes endothelial dysfunction, therefore increases CVD risk [27]. Many FA are associated, whereas others are inversely associated with CVD (Table 2). A strong inverse correlation was shown between RBC oleic acid and CVD [28]. Another study showed a protective effect of plasma very long-chain SFA (arachidic acid (20:0), behenic acid (22:0) and lignoceric acid (24:0)) in heart failure [29]. However, Hadj Ahmed et al. showed an increased level of RBC C26:0, C24:0, C22:0, EPA and AA in patients with coronary artery disease compared to the normal population and consider them biomarkers of coronary artery disease [9]. The same team showed an association between RBC and plasma trans FA and coronary artery disease severity [30]. Many other studies showed an association between RBC trans FA and coronary heart disease [26]. This association is mediated by increased low-density lipoprotein cholesterol and decreased high-density lipoprotein cholesterol [26]. Acute myocardial infarction is associated with decreased short-chain FA and increased long-chain FA levels [31]. Acute coronary syndrome is inversely associated with DHA/AA ratio [32]. Early onset coronary atherosclerosis is associated with decreased EPA and DHA levels [33] and risk of atherosclerotic plaque rupture is associated with high AA/DHA ratio [34]. The results are different for different studies. For example, cardiovascular disease is associated with elevated n-6 PUFA [35] or elevated SFA and trans FA [36] or increased MUFA [37] and the same is true for coronary artery diseases [9,38]. Such differences could be explained by genetic, lifestyle and dietary differences between the studied populations. One study showed that the association between FA and cardiometabolic risk is modulated by concurrent physical activity [39]. The main conclusion is that the FA profile is modified between healthy subjects and patients. The most consistent association is for increased trans FA and decreased DHA and EPA. 

Moreover, the FA profile could predict cardiovascular disease development in unhealthy subjects such as diabetic patients [40] and patients with renal failure [41]. Additionally, it predicts psychiatric disorder at 6 months after acute coronary syndrome [42]. Moreover, it predicts mortality among patients with acute cardiovascular disease [43], coronary artery disease [44] and with myocardial infarction [45].

#### 3.1.2. Fatty Acids and Diabetes (Table 2)

Many studies have highlighted the association between elevated total plasma free FA and diabetes [46,47,48], whereas other studies examined individual FA. RBC n-3 PUFA was negatively associated with the risk of type 2 diabetes [49]. Serum C22:0 and plasma n-6/n-3 were associated with diabetes [50,51]. Many studies have agreed on elevated plasma palmitic acid, stearic acid and oleic acid in diabetic patients [47,48,52,53,54,55]. In fact, palmitic acid slows down insulin signal transduction [56]. Meanwhile, the association between increased oleic acid concentration and the development of diabetes is not well understood, as studies show that oleic acid may play a role in the protection and treatment of diabetes [55]. Elevated RBC-linolenic acid was associated with high type 2 diabetes incidence [57]. Patel et al. showed that plasma FAs appear to be more strongly associated with diabetes incidence compared to RBC FA [58]. Serum EPA, C22:5 n-3 and DHA were associated with lower incidence of type 2 diabetes and higher insulin sensitivity [59]. Plasma trans FA are associated with diabetes through their association with fasting glucose, insulin, HbA1c and insulin index [60]. Moreover, the association between plasma trans FA (industrial and ruminant) and diabetes showed that ruminant trans FA (18:1 n-7 t and t10c12-CLA) are inversely associated with diabetes; however, c9t11-CLA is positively associated with diabetes [61]. In fact, the strength of the association between plasma FA and diabetes varied after adjustment for BMI, age and triglycerides [62]. Moreover, the n-6 to n-3 ratio was inversely related to diabetic retinopathy [63] and C22:0 was directly associated with a rapid decline in kidney function in T1D [64].

#### 3.1.3. Fatty Acids and Cancer (Table 2)

A study in patients with any type of cancer (except head and neck cancer) showed that cancer is associated with decreased SFA (C16:0 and C18:0) and increased MUFA (C18:1) and PUFA (LA and C20:3 n-6) [65]. Hepatocellular carcinoma is strongly associated with low levels of very long-chain SFA and n-3 PUFA [66]. Oral cancer is associated with decreased EPA and DHA [67]. Pancreatic cancer is associated with decreased n-3 PUFA and MUFA and increased arachidonic acid [68], whereas the association between FA and breast cancer depends on many variables, such as menopause [69] and BMI [70]. In breast cancer, FA (omega 3 and omega 6) modulate the cancer immune response and the fatty acid endogenous synthesis [71]. The concentration of oleic acid is lower in cancer patients, increasing their exposure to the disease [72]. In addition to their low level, an in vitro study showed that this fatty acid could be linked to cancer through the expression of miRNA [73]. Additionally, low RBC n-3/n-6 PUFA ratio was associated with multiple myeloma [74].

#### 3.1.4. Fatty Acids and Other Diseases (Table 2)

Serum γ-linolenic acid and C9:0 and C19:0 were associated with obesity [75,76] and dihomo-gamma-linolenic acid and palmitoleic acid were able to predict the future development of metabolic syndrome (MS) in obese subjects [77]. Zarrouk et al. identified hexacosanoic (C26:0) as blood (RBC and plasma) lipid biomarkers of dementia [7], whereas Yamagishi et al. showed that Serum ALA was inversely associated with the risk of disabling dementia [78]. Hammouda et al. showed that AA increased in plasma and RBC and DHA decreased only in plasma of Alzheimer patients [79]. Additionally, Sala-vila et al. found that DHA was inversely associated with Alzheimer’s disease [80]. Meanwhile, a study by Tomata et al. did not support the association between PUFA and Alzheimer’s disease risks [81]. Oresic et al. showed that decanoic and octanoic acids are associated with severe traumatic brain injury [82]. Zhao et al. found that PUFA are associated with liver injury [83] and Mutsuda et al. showed that Dihomo-γ-linolenic acid is associated with hepatic steatosis [84]. Two studies are in favor of the association between FA profile and autoimmune diseases [85,86]. Mikkelsen et al. showed that C22:5 n-3 and C18:1 n-9 can predict allergies in children [87]. Fares et al. mentioned that neonatal morbidities and mortality is associated with decreased AA and DHA [88].

**Table 2:** Fatty acid characteristics in many diseases.

Disease	Sample	Specific Fatty Acids	References
Cardiovascular diseases	Blood	Decreased n-6 PUFA	[35]
Cardiovascular disease	Blood	Increased SFA and trans FA	[36]
Cardiovascular risk	Serum	Increased MUFA Decreased n-6 PUFA and C22:6 n-3 (DHA)	[37]
Coronary artery disease	Red blood cells	Increased C26:0, C24:0, C22:0, C20:5 n-3 (EPA), C20:4 n-6 (AA) Decreased DHA, EPA/AA	[9]
Coronary artery disease	Plasma	Low levels of ALA, EPA, eicosatetraenoic (C20:4 n-3) and DHA	[38]
Coronary heart disease	Red blood cells	Increased Trans fatty acids	[26]
Coronary artery disease severity	Red Blood Cells and plasma	Increased Trans fatty acids	[30]
Heart Failure	Plasma	Decreased very long-chain saturated fatty acids arachidic acid (20:0), behenic acid (22:0) and lignoceric acid (24:0)	[29]
Acute myocardial infarction	Plasma	Decreased short-chain FA Increased long-chain FA	[31]
Acute coronary syndrome	Serum	Low DHA/AA	[32]
Early onset coronary atherosclerosis	RBC	Low EPA and DHA	[33]
Risk of atherosclerotic plaque rupture	serum	Increased AA/DHA	[34]
Type 2 diabetes	RBC	Elevated linolenic acid	[57]
Type 2 diabetes	Plasma	Increased palmitic, stearic and oleic acid	[55]
Type 2 diabetes	Plasma	Increased c9t11-CLA	[61]
Type 2 diabetes	Serum	Increased C22:6	[50]
Type 2 diabetes	Plasma	Increased n-6/n-3	[51]
Type 2 diabetes	Serum	EPA, C22:5 n-3 and DHA (inverse)	[59]
Cancer	RBC	Decreased SFA (C16:0 et C18:0) Increased MUFA (C18:1) Increased PUFA (LA and C20:3 n-6)	[65]
Oral cancer	RBC	Decreased EPA, DHA	[67]
Breast cancer	Plasma	Decreased linoleic acid (C18:2 n-6)	[72]
Pancreatic cancer	Plasma	Decreased n-3 PUFA and MUFA Increased AA	[68]
Hepatocellular carcinoma	Plasma	Very long-chain SFAs and very long-chain n-3 PUFAs (inverse)	[66]
Multiple myeloma	Red blood cell	Low n-3/n-6 PUFA ratio	[74]
Dementia	Red blood cells and plasma	Increased Hexacosanoic Acid (C26:0)	[7]
Dementia	Serum	ALA (inverse)	[78]
Alzheimer’s disease	Plasma and erythrocyte	Increased AA	[79]
	Plasma	Decreased DHA	
		The indexes AA/Dihomo-gamma-linolenic acid and C24:4 n-6/Adrenic acid (AdA) were both higher	
Alzheimer’s disease	Red blood cell	DHA (inverse)	[80]
Obesity	Serum	Increased γ-linolenic acid	[75]
Obesity	Serum	Increased C9:0 and C19:0	[76]
Drug-induced liver injury	Serum	Altered PUFA	[83]
Hepatic steatosis	Serum	Increased dihomo-γ-linolenic acid	[84]
Severe traumatic brain injury	Plasma	Increased decanoic and octanoic acids	[82]
Neonatal morbidities and mortality	Red blood cell	Low AA and DHA	[88]
Autoimmune diseases	Serum	decreased n-6/n-3 ratio	[85]
Autoimmunity	Red blood cell	Decreased levels of n-3 fatty acid and docosapentaenoic acid	[86]
Allergy in children	Blood	Increased C22:5 n-3 and decreased C18:1 n-9	[87]

### 3.2. Polymorphisms of Gene Implicated in Fatty Acid Metabolism and Diseases

**Figure 1:**
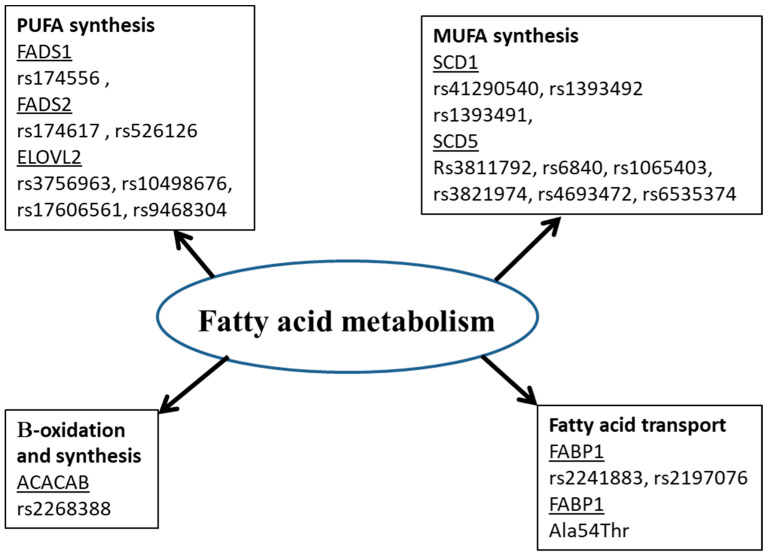
Polymorphisms of genes for proteins involved in fatty acid metabolism. ACACB: Acetyl-coenzyme A carboxylase, ELOVL: Elongase, FADS: Fatty Acid Desaturase, FABP: Fatty Acid Binding Protein, MUFA: monounsaturated fatty acids, PUFA: polyunsaturated fatty acids, SCD: stearoyl Co A desaturase.

Many studies showed that the fatty acid in biological tissues is an indicator of fatty acid intake [89,90]. However, this is not always the case. Amézaga et al. showed that the variation in erythrocyte FA is not linked to dietary intake [65]. FA have an exogenous and endogenous origin. They can be provided by food or synthesized, mainly in hepatocytes. Several proteins are involved in the metabolism of FA (Figure 1). Any perturbation in the activity or quantity of those enzymes could be a cause of a fatty acid profile change, then disease development [5]. Enzyme activity and expression are under genetic and environmental control. In this paper, we try to summarize the contributions of some genetic polymorphisms of many proteins involved in fatty acid metabolism (FADS, ELOVL, Fatty acid binding protein and acetyl-coenzyme A carboxylase) to the development of certain diseases (Table 3).

Delta 9 MUFAs (mainly oleic acid) are synthesized under the control of many enzymes. The key enzyme of desaturation is stearoyl Co A desaturase (SCD: SCD1 and SCD5). This enzyme plays a key role in the development of many diseases. Thus, inhibiting it will have many beneficial effects. SCD1 enzyme was a potential target for cancer [91], non-alcoholic fatty liver disease [92], diabetes, obesity and hepatic steatosis [93]. The activity of SCD1 is usually calculated as C18:1/C18:0 ratio. Single Nucleotide Polymorphisms (SNP) in SCD1 gene (located on chromosome 10q24.31) are associated with many diseases. The rs41290540 SNP in the 3’-untranslated region is associated with decreased risk of coronary artery disease [94]. Liu explained this association based on the fact that this SNP increases SCD1 expression (in vitro study), and then the concentration of MUFA will increase and reduce total cholesterol concentration. Another SNP located in the 3’-untranslated region, the rs 1393492, is associated with metabolic syndrome in dialysis patients [95]. Guo et al. showed that AA genotype is associated with a large waist circumference, high blood pressure and high blood glucose. Another polymorphism (rs1393491) in SCD1 gene is associated with Grove’s ophthalmology [96].

The association between SCD1 gene polymorphism and disease development depends on many factors, such as diet: oil intake in case of obesity [97] and PUFA intake in case of cancer death [98]. The relationship between SCD1 and disease development could be explained by the variation in SCD1 expression according to gene variant, which influences the fatty acid profile (unsaturated: saturated fatty acid ratio) [99]. Despite the great attention attributed to SCD1 isoform, few studies on SCD5 isoform have been conducted. The rs3811792 SNP in SCD5 promoter is associated with type 1 and type 2 diabetes [100]. This polymorphism decreases SCD5 promoter activity. Zambo explained the association between the polymorphism and type 2 diabetes based on the fact that SCD5 regulates the distribution of fats and accumulation in viscus, which represents a diabetes risk factor. However, the association with type 1 diabetes is due to the overexpression of SCD5 in the pancreas compared to other tissues. Many other polymorphisms in SCD5 gene are described: rs6840, rs1065403, rs3821974 in 3′-UTR and rs4693472, rs6535374 in intron. All of them are associated with hepatocellular carcinoma [101].

**Figure 2:**
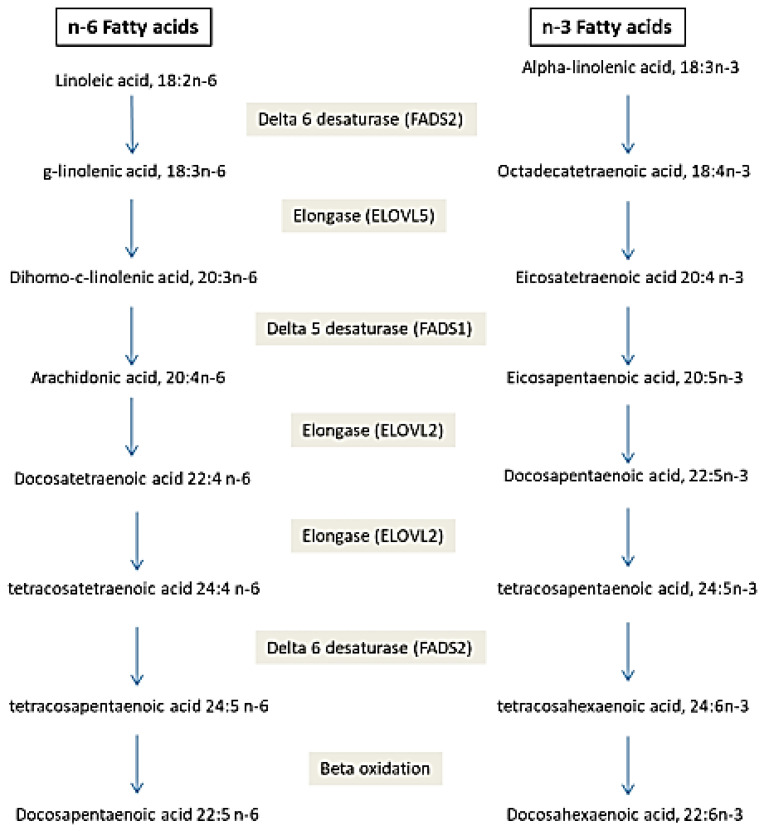
Desaturase and elongase metabolic pathway in long-chain fatty acid synthesis.

PUFA synthesis requires other enzymes. Deasaturase and elongase enzymes involved in fatty acid synthesis from ALA and LA are summarized in Figure 2. Two types of desaturases exist: delta-6-desaturase (FA desaturase 2, FADS2) and delta5-desaturase (FA desaturase 1, FADS1). FADS2 converts substrates ALA and LA, respectively at C18:4 n-3 and C18:3 n-6. This enzyme is also involved in a second step leading to the desaturation of the C24:5 n-3 and C24:4 n-6 substrates to C24:6 n-3 and C24:5 n-6, respectively. FADS1 desaturated the FA C20:4 n-3 and C20:3 n-6, respectively into EPA and AA. The activity of elongases (ELOVL) on eighteen- and twenty-carbon FA and n-3 long-chain PUFA is the result of ELOVL5 gene expression. On the other hand, ELOVL2 is the gene allowing the elongation of PUFAs with twenty and twenty-two carbon atoms. The genes coding for desaturases and elongases are the most studied to explore the genetic effect of FA on the development of diseases [79,102,103,104]. Hammouda et al. showed that rs174556 TT genotype of FADS1 is associated with a higher AA level and AA/DGLA (Dihomo-gamma-linolenic acid) index and increased risk of Alzheimer’s disease. Additionally, rs3756963 TT genotype of ELOVL2 is associated with increased AA and AA/DGLA index and increased risk of Alzheimer’s disease. However, rs174617 of FADS2 is not associated with either the fatty acid profile or Alzheimer’s disease. The combination of the two variants increases further the susceptibility to Alzheimer’s disease.

This study [79] suggested that FADS1 and ELOVL2 variants could likely influence the AA biosynthesis, then the inflammation. Khamlaoui et al. studied the association between gene polymorphisms and obesity [103]. They showed that rs174556 C allele of FADS1, rs174617 of FADS2 and rs3756963 of ELOVL2 are risk factors of obesity. The two polymorphisms rs174556 of FADS1 and rs174617 of FADS2 are, respectively, associated with low DHA and High EPA. However, the association between ELOVL2 and FA was unclear. The above two studies suggest that the association between gene polymorphism and fatty acid profile could, in part, explain the association between those polymorphisms and disease development. Song et al. showed an association between rs174556 of FADS1 and Acute Coronary Syndrome and no association for FADS2 and ELOVL2 polymorphisms [102]. Sun et al. showed an association between rs526126 of FADS2, rs10498676, rs17606561, rs3756963 and rs9468304 of ELOVL2 and Autism spectrum disorder [104]. However, in the two cited studies, no analysis of fatty acid was performed. Sun et al. explained that FADS polymorphisms may cause irreversible functional and structural changes in neurons by acting on FA, which can lead to Autism spectrum disorder.

The fatty acid-binding protein (FABP) family includes several FABPs that are abundantly expressed in tissues with active fatty acid metabolism. FABPs would have several roles, including the intracellular transport of fatty acid chains and other lipophilic substances, such as eicosanoids and retinoids, from plasma membranes to sites of metabolism. Members of this family include FABP1 in liver, FABP2 in intestine, FABP3 in muscle and heart and FABP4 in adipocytes. Valizadeh et al. showed that the rs2241883 CC genotype of FABP1 gene is associated with dyslipidemia [105] and Xue et al. found that rs2197076 and rs2241883 of the same protein are associated with polycystic ovary syndrome [106]. According to Xue et al., rs2197076 seemed to have a more important role in the mechanism of polycystic ovary syndrome than rs2241883 because it is closely related to some important clinical features of polycystic ovary syndrome. Moreover, rs2197076 FABP1 is associated with type 2 diabetes [107], rs2241883 is associated with metabolic syndrome [108] and rs2241883 with rs1545224 are associated with non-alcoholic fatty liver [109]. The most FABP-studied polymorphism is located in FABP2 gene. It is an amino acid substitution (Ala54 to Thr54). This polymorphism is associated with diabetes, metabolic syndrome, obesity and non-alcoholic liver disease [110,111,112,113]. In diabetic patients, the polymorphism is associated with peripheral atherosclerosis, retinopathy and renal disease in diabetic patients [114,115,116]. Another polymorphism in FABP2 is associated with essential hypertension [117] and diabetes [118].

Acetyl-coenzyme A carboxylase (ACACB) catalyzes the synthesis of malonyl-CoA, a metabolite that plays an essential role in the synthesis and oxidation of FA. A meta-analysis showed that rs2268388 C allele in ACACB was inversely associated with susceptibility risk of diabetic nephropathy among Caucasian patients [119], Chinese patients [120] and Asian Indian patients [121]. However, Chan et al. did not find any association between ACACB gene polymorphism and cardiovascular risk susceptibility in type 2 diabetic patients [122]. In Spanish postmenopausal women, the rs2268388 T allele was associated with obesity and diabetes and the rs2239607 C allele was associated with diabetes [123]. Additionally, another polymorphism (rs47665887) in ACACB gene influence metabolic syndrome, but this association is modulated by dietary fat [124].

Genetics alone cannot confirm the presence of a disease, except in certain cases in which polymorphisms are mutations that directly affect the presence or function of the enzyme. However, genetic studies make it possible to predict diseases and help to manage them. In fact, diseases are usually multifactorial. One disease can be the result of many protein disturbances. Additionally, environmental factors such as diet, temperature and stress can modulate the effect of genetic factors.

## 4. Conclusions

FA and the genes involved in their metabolism can be considered predictors of certain disease development and progression. However, in many cases, the results are inconsistent. Additional studies should be considered to better highlight the factors that may influence these associations in order to finally define powerful predictors.

## Figures and Tables

**Table 1:** Fatty acid profile in three different studies.

	Study 1 [6]		Study 2 [7]		Study 3 [8]	
Samples	Plasma	RBC	Plasma	RBC	Plasma	RBC
Total SFA	29.9 ± 2.3	42.1 ± 4.6	34.22 ± 3.26	50.56 ± 6.82	28.71 ± 2.38	34.37 ± 1.60
Lauric acid, 12:0	0.09 ± 0.02	-	-	-	0.02 ± 0.03	0.002 ±0.01
Myristic acid, 14:0	0.95 ± 0.3	0.73 ± 0.2	-	-	0.58 ± 0.28	0.19 ± 0.10
Palmitic acid, 16:0	21.1 ± 1.9	25.9 ± 3.0	26.24 ± 2.75	30.05 ± 4.71	19.31 ± 2.41	18.65 ± 1.86
Stearic acid, 18:0	7.08 ± 0.8	13.2 ± 1.2	7.16 ± 1.52	16.51 ± 3.41	7.29 ± 0.78	13.14 ± 1.00
Arachidic acid, 20:0	0.06 ± 0.05	0.14 ± 0.1	0.25 ± 0.35	0.62 ± 0.27	-	-
Behenic acid, 22:0	0.19 ± 0.07	0.50 ± 0.2	0.30 ± 0.12	1.71 ± 0.62	-	-
Lignoceric acid, 24:0	0.17 ± 0.08	1.71 ± 0.7	0.17 ± 0.05	0.79 ± 0.29	-	-
Cerotic acid, 26:0	-	-	0.1 ± 0.05	0.88 ± 0.06	-	-
Total MUFA	25.0 ± 4.3	20.8 ± 2.9	28.2 ± 3.48	30.71 ± 5.13	22.86 ± 3.01	18.64 ± 1.24
Myristoleic acid, 14:1 n-5	0.01 ± 0.01	0.03 ± 0.1	1.32 ± 1.21	1.52 ± 1.23	-	-
Palmitoleic acid, 16:1 n-5	-	-	2.53 ± 0.9	1.53 ± 0.97	-	-
Oleic acid, 18:1 n-9	22.7 ± 3.4	18.4 ± 2.4	21.01 ± 3.56	22.01 ± 4.62	18.60 ± 2.43	13.26 ± 1.17
cis-Vaccenic acid, 18:1 n-7	-	-	1.93 ± 0.48	2.41 ± 0.68	-	-
Eicosenoic acid, 20:1 n-9	0.25 ± 0.1	0.90 ± 0.2	0.64 ± 0.13	0.49 ± 0.29	-	-
Erucic acid, 22:1 n-9	-	-	0.43 ± 0.16	1.33 ± 1.05	-	-
Nervonic acid, 24:1 n-9	-	-	0.34 ± 0.02	1.42 ± 1.08	-	-
Total PUFA	45.1 ± 4.9	37.1 ± 6.7	37.58 ± 4.67	18.73 ± 4.97	44.87 ± 4.81	43.48 ± 1.77
Alpha-linolenic acid, 18:3 n-3	0.69 ± 0.1	0.12 ± 0.01	0.71 ± 0.23	0.47 ± 0.47	0.50 ± 0.15	0.18 ± 0.05
Eicosatrienoic acid, 20:3 n-3	-	-	1.75 ± 0.48	1.71 ± 0.98	-	-
Eicosapentaenoic acid, 20:5 n-3	0.50 ± 0.2	0.32 ± 0.2	0.30 ± 0.31	0.61 ± 0.41	0.49 ± 0.21	1.15 ± 0.91
Docosahexaenoic acid, 22:6 n-3	-	-	1.43 ± 0.27	0.73 ± 0.07	1.56 ± 0.60	3.71 ± 1.09
Linoleic acid, 18:2 n-6	33.7 ± 4.5	14.8 ± 2.2	27.78 ± 5.26	9.49 ± 3.31	30.58 ± 4.33	13.66 ± 1.80
linolenic acid, 18:3 n-6	0.36 ± 0.1	0.40 ± 0.2	0.59 ± 0.30	0.39 ± 0.45	-	-
Dihomo-c-linolenic acid, 20:3 n-6	1.29 ± 0.3	1.50 ± 0.4	0.57 ± 0.37	2.01 ± 0.85	-	-
Arachidonic acid, 20:4 n-6	6.27 ± 1.6	4.82 ± 1.52	4.45 ± 1.24	3.32 ± 2.06	7.80 ± 1.62	14.63 ± 1.29

**Table 3:** Association between genetic polymorphisms, fatty acid characteristics and diseases.

Genes	Polymorphisms	Diseases	References
SCD1	rs41290540	Decreased risk of coronary artery disease	[62]
	rs1393492	Metabolic syndrome	[63]
	rs1393491	Graves’ Ophthalmopathy	[64]
SCD5	rs3811792	Diabetes	[68]
	rs6840, rs1065403, rs3821974, rs4693472, rs6535374	Hepatocellular carcinoma	[69]
FADS1	rs174556	Increase the risk of Alzheimer’s disease	[53]
		Acute Coronary Syndrome	[70]
		Obesity	[71]
FADS2	rs174617	No effect on the risk of Alzheimer’s disease	[53]
		obesity	[71]
	rs526126	Autism spectrum disorder	[72]
ELOVL2	rs3756963	Increase the risk of Alzheimer’s disease	[53]
		obesity	[71]
	rs10498676, rs17606561, rs3756963 and rs9468304	Autism spectrum disorder	[72]
FABP1	rs2241883	Dyslipidemia	[73]
	rs2197076 and rs2241883	Polycystic ovary syndrome	[74]
	rs2241883 and rs1545224	Non-alcoholic fatty liver	[77]
	rs2197076	Type 2 diabetes	[75]
	rs2241883	Metabolic syndrome	[76]
FABP2	Ala54Thr	Diabetes, metabolic syndrome and obesity	[78]
		Peripheral atherosclerosis combined with T2DM	[82]
		Obesity	[79]
		Diabetic retinopathy	[83]
		Type 2 diabetes and metabolic syndrome	[80]
		Renal disease in type 2 diabetic	[84]
		Non-alcoholic fatty liver	[81]
	rs1799883	Essential hypertension	[85]
		Diabetes	[86]
ACACB	rs2268388	Diabetes related nephropathy	[87,89]

## Data Availability

Not applicable.
